# The role of microbiomes in gastrointestinal cancers: new insights

**DOI:** 10.3389/fonc.2023.1344328

**Published:** 2024-02-01

**Authors:** Aref Yarahmadi, Hamed Afkhami

**Affiliations:** ^1^Department of Biology, Khorramabad Branch, Islamic Azad University, Khorramabad, Iran; ^2^Nervous System Stem Cells Research Center, Semnan University of Medical Sciences, Semnan, Iran; ^3^Cellular and Molecular Research Center, Qom University of Medical Sciences, Qom, Iran; ^4^Department of Medical Microbiology, Faculty of Medicine, Shahed University, Tehran, Iran

**Keywords:** gastrointestinal cancer, gut microbiome, microbiota, cancer therapy, inflammation

## Abstract

Gastrointestinal (GI) cancers constitute more than 33% of new cancer cases worldwide and pose a considerable burden on public health. There exists a growing body of evidence that has systematically recorded an upward trajectory in GI malignancies within the last 5 to 10 years, thus presenting a formidable menace to the health of the human population. The perturbations in GI microbiota may have a noteworthy influence on the advancement of GI cancers; however, the precise mechanisms behind this association are still not comprehensively understood. Some bacteria have been observed to support cancer development, while others seem to provide a safeguard against it. Recent studies have indicated that alterations in the composition and abundance of microbiomes could be associated with the progression of various GI cancers, such as colorectal, gastric, hepatic, and esophageal cancers. Within this comprehensive analysis, we examine the significance of microbiomes, particularly those located in the intestines, in GI cancers. Furthermore, we explore the impact of microbiomes on various treatment modalities for GI cancer, including chemotherapy, immunotherapy, and radiotherapy. Additionally, we delve into the intricate mechanisms through which intestinal microbes influence the efficacy of GI cancer treatments.

## Graphical Abstract

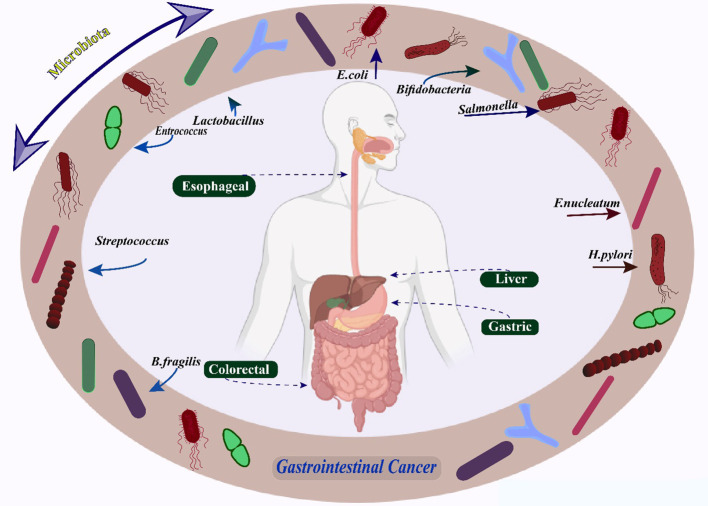

## Introduction

Gastrointestinal (GI) malignancies constitute approximately one-third of all newly diagnosed cancer cases globally and pose a significant public health challenge. Colorectal cancer (CRC), Gastric cancer (GC), liver cancer, and esophageal cancer are the most commonly observed GI malignancies across the globe (1, 2). Since GI malignancies have been on the rise over the past 5 to 10 years, there is a severe health risk to people due to this trend. The past decade has witnessed the substantiation of the role played by genetic and epigenetic factors in the development of cancer. This has been achieved through the extensive genomic and transcriptome sequencing endeavors undertaken by multiple multinational research initiatives (3). According to current studies, 2.7 million individuals worldwide die from GI cancer yearly, with 4 million cases diagnosed worldwide (4–6). Although GI cancers display a diverse array of biological attributes, several shared risk factors have been discerned. These include pro-tumor genetic mutations, excessive intake of alcohol, smoking, adherence to the Western diet, exposure to radioactive stimuli, and disturbance of the GI microbiota’s homeostasis (7). Furthermore, the disruption of the typical GI environment has been associated with the onset of GI malignancies due to the emergence of pro-tumoral fibrosis and the occurrence of significantly potent local or systemic inflammatory and immunological reactions (8–10). In addition to these risk factors, certain disorders are strongly linked to the origin of GI cancers. For instance, it has been found that GI cancer and diabetes are related. One of the most used anti-hyperglycemic medications, metformin, has been demonstrated to lower the incidence rate of GI malignancies in diabetic individuals (11, 12). The comprehension of the role of bacteria in cancer development is significantly restricted compared to the knowledge we have about viruses causing oncogenesis. However, it is feasible to consider that gaining a better understanding of the long-lasting effects of changes in the composition of the GI microbiota may have the potential to contribute to the progress of preventive strategies against cancer. Moreover, bacteria have the potential to indirectly facilitate the process of carcinogenesis through the alteration of both systemic and local immune reactions. These immune responses play a crucial role in progressing GI tract malignancies (13). The GI tract of humans harbors a vast number of microorganisms that work in conjunction with the host to uphold both wellness and illness. The intricate web of interactions between the GI microbiome and the host gives rise to intimate connections that span various components of human physiology, such as the metabolic, immunological, and neuroendocrine systems (14). These creatures are dynamic and subject to influences from medications, food, lifestyle, genetics, and the environment (15). Researchers have discovered that the influence of gut microorganisms extends beyond the confines of the intestines, affecting a range of conditions including pancreatic disease and hepatic disease, in addition to intestinal diseases such as Inflammatory bowel disease (IBD) and CRC (16). Shortly following the moment of birth, the microbiota initiates the process of establishing residence within the GI tract, subsequently maintaining its presence throughout the entirety of an individual’s lifespan (17). However, it can vary dynamically in response to nutrition, environmental stresses, lifestyle choices, antibiotics, and other medications (18). Increasing proof suggests that the microbial population residing in the digestive system holds immense potential to thwart the growth of cancerous cells while also possessing the ability to enhance the potency of chemotherapy and immunotherapy treatments (19). The gut microbiota is accountable for producing short-chain fatty acids (SCFAs), which bestow advantageous effects on the human body. These SCFAs are generated through the metabolic breakdown of dietary fiber, as well as the synthesis of vitamins B and K2. Additionally, the gut microbiota metabolizes various chemicals, such as sterols and exogenous substances, while also playing a role in regulating immunological function (20).

This study aimes to investigate the primary impacts of intestinal microbiota on the initiation and advancement of GI cancers, along with the potential utilization of these microorganisms as a sophisticated approach to discern and manage these ailments, as expounded upon in this comprehensive analysis.

### Microbiome in health and gastrointestinal cancer

The analysis of the microbial populations found in various human environments, such as the GI tract, mouth, skin, and vaginal area, requires applying advanced sequencing techniques that can process large amounts of data (21). The utilization of sophisticated sequencing methodologies, encompassing amplicon and shotgun metagenomic sequencing, has significantly transformed our comprehension of the human microbiome by delineating the bacteria linked to either optimal well-being or pathological conditions (22). When the physical condition of an individual is in a state of good health, the gut microbiota engages continuously and regularly with the host organism to sustain a state of balance within the intestines (23). However, maintaining such balance is challenging since the host’s genetic makeup and several exogenous variables, including nutritional consumption and antibiotic usage, have a direct impact on the microbiome (24–26). Dysbiosis, the alteration in both composition and functionality within the microbiome, can occur when there is a persistent disturbance in the stability of the microbial community. This alteration may cause various disorders, including cancer (27, 28). In a dysbiotic microbiome, various pathogenic occurrences are encountered, including a modification in the assortment of microorganisms, a decrease in beneficial commensals, and the proliferation of pathobionts. All of these occurrences can impact the formation of tumors, either in the vicinity of the GI tract or at a more remote location, such as the pancreas and liver (29, 30). The GI system harbors the highest abundance of commensal microorganisms among all the regions of the human body. Variable parts of the digestive system have varying levels of commensal microbial diversity and abundance (31). While a multitude of bacteria belonging to the phyla *Firmicutes*, *Proteobacteria, Actinobacteria*, *Firmicutes*, *Bacteroidetes*, and are frequently observed within the gut, certain bacterial species seem to be confined to specific regions (31, 32). Each area or organ’s microbial population is related to host characteristics, including pH, oxygen saturation, bile acids, and nutritional bioavailability (33, 34).

### GI cancer and gut microbiota

The thorough analysis of microbial populations in the host’s environment has been extensively explained as a result of the rapid advancements achieved in next-generation high-throughput sequencing (NGS) (35, 36). Dysbiosis leads to the stimulation of inflammatory components within the GI mucous membranes, which encompasses the liberation of nitric oxide (NO), the presence of oxidative stress, the creation and excretion of pro-inflammatory cytokines, as well as the activation of cyclooxygenase 2 (COX-2). Dysbiosis also causes microecological alterations (27, 37). The detrimental effects of microbial metabolites on extra-intestinal organs can manifest in various pathways, such as the gut-liver axis and the gut-brain axis, thereby impairing their optimal functioning (38, 39). Dysbiosis is believed to be most comprehensible when viewed through carcinogenesis, representing a continuous divergence of the host microbiota from a state of harmony and equilibrium that supports and, or upholds various cancer phenotypes (40, 41). The maintenance of well-balanced gut microbiota is crucial for promoting a healthy lifestyle. At the same time, an imbalance in this microbial community, known as dysbiosis, can lead to inflammatory consequences that accelerate cancer progression (42).

### The role of the microbiome in colorectal cancer

Despite an increasingly widespread acceptance of colonoscopy screening, colorectal cancer (CRC) remains the third most commonly occurring cancer and the primary contributor to cancer-related deaths in both male and female populations within the United States (43). In 2019, a forecast indicated that there would be an estimated 51,020 deaths and approximately 145,600 fresh instances of CRC. Additionally, while the occurrence and fatality rate of CRC has experienced a gradual decrease in the past few decades in individuals aged 65 and above, a distinct trend has emerged among individuals under 50, for whom conventional screening methods have not been recommended (44). The evolution of CRC has been comprehensively examined over recent decades via migration and prospective cohort studies, illustrating the significant influence of nutritional and lifestyle determinants (43). According to estimations, it has been noted that modifiable risk factors, namely excessive weight or obesity, excessive alcohol consumption, smoking, high consumption of red meat, physical inactivity, and inadequate intake of dietary fiber, whole grains, or other beneficial nutrients, play a significant role in approximately 50% to 60% of newly reported cases of CRC in the US (43). The microbiome, which encompasses bacteria, viruses, fungi, and an array of diverse organisms, possesses the ability to regulate the condition of well-being, and modifications to it can contribute to the emergence and progression of ailments. There exists an increasing corpus of scholarly investigation indicating that alterations in the constitution of the intestinal microbiota contribute to the genesis and progression of CRC using the impact of environmental factors that pose a risk (45, 46). This could be the case due to the microbiome’s effect on metabolism and immune response (47). Hence, manipulating the intestinal microbial community could potentially serve as a constituent of strategies aimed at averting CRC (48, 49). Research has demonstrated discrepancies in the composition of the intestinal microbiomes between individuals afflicted with CRC and those deemed healthy (controls). Additionally, certain microbial species have been identified as exhibiting increased or diminished presence within the gut microbiomes of CRC patients. Therefore, to improve screening techniques, alterations in the microbiome can be utilized as biomarkers in the early detection of CRC (49). The colon is home to 70% of the human microbiome (50). Individuals who encounter antibiotics early on exhibit an increased propensity to develop colorectal adenoma during their later years (51). The microbiota in the GI tract plays a crucial role in converting the dietary components into metabolites that can either promote or suppress the growth of tumors. The development of CRC is subsequently influenced by these metabolites (52). Over 2000 different bacteria species are thought to exist in the human gut (53, 54).

### Microbes associated with risk of CRC

#### Fusobacterium nucleatum

**Figure 1:**
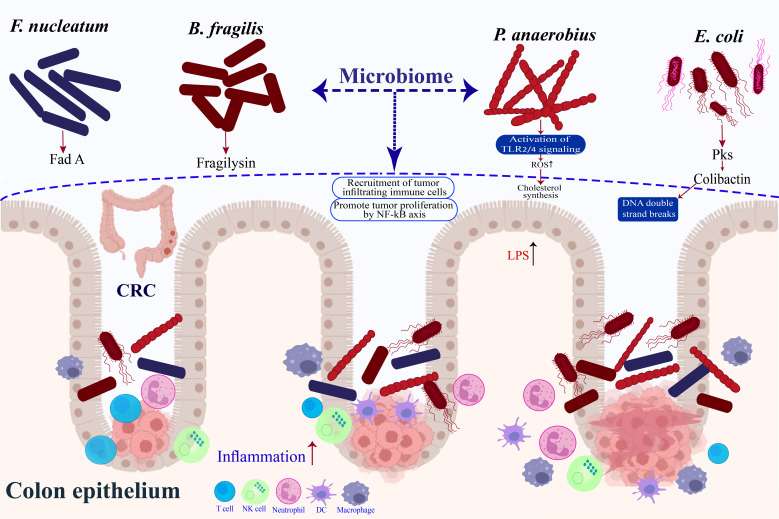
Microbes associated with risk of colorectal cancer.

According to two separate investigations, tumor specimens had more *Fusobacterium* DNA and RNA sequences than non-tumor ones (55, 56). Similar relationships have been discovered in several studies, including numerous cohorts of CRC patients worldwide (57, 58). *Fusobacterium nucleatum* (*F. nucleatum*) has been associated with more advanced stages of disease, an increased likelihood of recurrence, and shorter periods of survival for patients, thus presenting compelling evidence of its potential causal role in CRC (59) (**Figure 1**). It is found in 10%–15% of tumors. Furthermore, *F. nucleatum* levels in tumor tissue have been linked to reduced T-cell infiltration, corroborating studies that claim *F. nucleatum* inhibits the anti-tumor immune response (43). *F. nucleatum* has been linked to distinct clinical and molecular characteristics in epidemiological investigations involving individuals with CRC or precancerous lesions. The aforementioned characteristics encompass the presence of anatomical positioning on the right side, mutations in the BRAF gene, and heightened levels of hypermutation alongside microsatellite instability (58, 60, 61). The described attributes of serrated neoplasia imply that *F. nucleatum* might have a part to play in developing CRC through the serrated pathway. Research has established a connection between *F. nucleatum* and the consensus molecular subtype 1 of CRC (62). This particular subtype is distinguished by an excessive expression of the immune system and the presence of microsatellite instability (63–65). More recently, in paired main tumors and distant metastases from CRC patients, virtually identical, live *Fusobacterium* strains were discovered in similar relative abundances. *Fusobacterium* thus seems to be a crucial part of the tumor microenvironment (66).

#### Bacteroides fragilis

Enterotoxigenic *Bacteroides fragilis* (ETBF), a significant pathogen that releases virulence factors to advance CRC, generates *B. fragilis* toxin or fragilysin, thereby inciting an adverse immune reaction (67) (**Figure 1**). Colitis characterized by a robust, selective colonic Stat3 activation and a selective Th17 response was seen in mice treated with ETBF (68). Notably, a study by Chung et al. (69) provided more evidence that BFTs focus on the epithelial cells of the colon to instigate an immune response within the mucosal lining. This, in turn, triggers a series of inflammatory reactions that require the activation of IL-17, NF-kB, and Stat3. The molecular pathways of ETBF-induced adaptive immunity modification in CRC were further characterized by other researchers. Geis and colleagues (70) demonstrated that the presence of regulatory T cells in the local microenvironment resulted in a decrease in the quantity of IL2, thereby enabling the proliferation of Th17 cells, which is essential for the promotion of ETBF-induced CRC. Exosome miR-149-3p produced by colon cells after ETBF treatment also promotes Th17 differentiation (67). Consequences of long-term ETBF infection and inflammation include carcinogenesis. According to an animal study, BFT was required for the impact of ETBF infection, which increased colonic inflammation and enhanced AOM/DSS-induced CRC (71). An IL-17-driven monocytic-MDSC-dominant immunological profile was shown to be related to ETBF-triggered CRC by Thiele et al. (72), indicating that ETBF infection encourages MDSC-mediated immune suppression*. B. fragilis* emerged as the sole species consistently exhibiting higher levels in the intestinal microbiomes of individuals diagnosed with CRC across various geographical regions. This finding was established through a comprehensive meta-analysis encompassing four case-control studies that examined the metagenomes of CRC patients (73).

#### Escherichia coli

There is growing proof that pks+ *Escherichia coli (E. coli)* can produce virulence factors that control the development and progression of CRC (74) (**Figure 1**). A cancer-related pathogen that often infects CRC patients and expresses the polyketide synthase (pks) gene Colibactin, a hybrid peptide-polyketide cytotoxin that *E. coli* produces, induces DNA double-strand breaks and activates the DNA damage checkpoint mechanism in eukaryotic cells (75, 76). The involvement of colibactin in CRC has been shown by recent research. Anaphase bridge development, G2/M cell cycle stoppage, chromosomal aberration, and instability are all signs of the DNA damage response that even brief exposure to pks+ *E. coli* causes in mammalian epithelial cells (76–78). Cougnoux and colleagues (79), on the other hand, showed that acceleration of AOM-DSS-induced CRC by pks+ *E. coli* is facilitated by the stimulation of growth factor-secreting senescent cells, which is achieved through the alteration of p53 SUMOylation. Consequently, this modification encourages the proliferation of uninfected cells. Colibactin-producing *E. coli* also alters the immunological milieu, decreasing antitumor T-cell response and causing immunotherapy resistance and their effects on DNA damage (80).

#### Peptostreptococcus anaerobius

An anerobic bacteria called *Peptostreptococcus >anaerobius* (*P. anaerobius*) often lives in the mouth cavity. The bacterium *P. anaerobius*, which has been recently identified, was observed to have a higher occurrence rate among individuals diagnosed with CRC in comparison to those who were in good health (81, 82) (**Figure 1**). This remarkable finding was unearthed by employing the cutting-edge technique of shotgun metagenomic sequencing on fecal samples coupled with the exact 16S ribosomal RNA sequencing method on mucosal samples (83, 84). Subsequent examinations of functional nature revealed that *P. anaerobius* expedited the progression of AOM-induced CRC by augmenting the synthesis of cholesterol through the activation of TLR2/TLR4 signaling, thereby bolstering the proliferation of CRC cells (85, 86). Moreover, investigations on the profiling of the microbiome in the oral cavity have revealed that there are variations in the quantities of different components of the oral biofilm, including *Parvimonas*, *Haemophilus*, *Alloprevotella*, *Prevotella*, *Lachnoanaerobaculum*, *Streptococcus*, and *Neisseria*, between patients with CRC and control subjects (87, 88). The development of CRC has been associated with varying gene expression patterns in the mucosal surfaces of different bacteria. Notably, inquiries that have examined samples derived from individuals with colonic neoplasia and controls have discovered analogous networks of oral bacteria that exist on both the oral and colonic mucosal surfaces (88).

### Modulation of microbiota in CRC

In light of the crucial function that the gut microbiota fulfills in CRC, extensive investigations have been conducted to unravel the secrets of regulating gut dysbiosis to avert or combat this ailment (89). Several tactics have been suggested, such as fecal microbial transplantation (FMT), dietary changes, and antibiotic treatment. At present, FMT has demonstrated efficacy in the management of recurrent *Clostridium difficile* infection. Nevertheless, the utilization of FMT in animals remains limited to the prevention and treatment of CRC (90–94). FMT is only marginally beneficial in a preventative situation, though. A more likely method of controlling the microbiota to prevent CRC is dietary intervention. Recent research has revealed how food and the microbiota interact to cause CRC (95). For instance, high-fat diet-fed mice showed a considerable change in the makeup of their intestinal microbial and reduced gut barrier function, confirming the theory that high fat causes CRC by encouraging microbial dysbiosis (96). Contrarily, dietary fiber can promote the proliferation of advantageous commensals, which produce metabolites such as Short-chain fatty acids (SCFAs) linked to tumor-suppressing properties (97). Intriguingly, ETBF-induced CRC in AOM-DSS mouse models was suppressed by high salt diets, suggesting that the effect of diet may depend on the situation (98, 99).

### Effect of microbiota on cancer therapy in CRC

Current research suggests that the makeup of microorganisms in the GI system, referred to as the intestinal microbiome, can impact the body’s response to different cancer therapies, including immune checkpoint blockade (ICB) therapy and chemotherapy. The study involved comparing samples of rectal cancer that were locally advanced, with and without treatment for *F. nucleatum*. The results showed that *F. nucleatum* persistence after neoadjuvant chemoradiotherapy (nCRT) is connected to increased recurrence rates and the inhibition of immune cytotoxicity (100). Time and time again, numerous studies have discovered that *F. nucleatum* was more commonly present in patients with CRC who experienced a recurrence following chemotherapy (101). Additionally, it was observed that *F. nucleatum* targeted microRNAs, as well as the innate immunological signaling pathways TLR4 and MYD88, to stimulate the defense autophagy pathway and counteract the response to chemotherapy (102, 103). These findings imply that pathogenic microorganisms may not only influence colorectal carcinogenesis but also enhance treatment resistance (104). Contrarily, much research has also surfaced, demonstrating that gut commensal bacteria can enhance ICB treatment by activating antitumor T cells (105–107).

## The role of the microbiome in gastric cancer

Gastric cancer (GC) holds a prominent position as the fourth leading contributor to cancer-related mortality globally. Furthermore, it also ranks as the fifth most commonly detected form of cancer (108). Male rates are two times higher than female rates. Eastern Asia has the highest incidence rates, approximately 26,000 fresh instances and 11,000 fatalities from GC manifest annually within the US. The overall 5-year survival rate for GC is considerably low, standing at 32.4%. This is probably because, in the United States, up to 62% of instances of GC are diagnosed at late stages, which are linked to lower overall survival rates than localized illness (109). GC arises as a result of a multifaceted interplay involving the genetic composition of the host, various environmental factors (e.g., alcohol consumption, smoking, excessive intake of salt and meat, and inadequate consumption of vegetables and fruits), as well as microbial elements (e.g., the presence of *Helicobacter pylori* (*H. pylori*) infection and the composition of the intestinal microbiota). The persistent activation of the immune system resulting from the intestinal microbiota of the host has been associated with long-term inflammation and altered interactions between the host’s epithelium and microorganisms, which have been associated with GC (110).

### Microbiota in the healthy, non-neoplastic stomach

The standard oxyntic (corpus) region of the human stomach, characterized by a low pH and an acidic milieu, is a barrier against the proliferation of commensal organisms and potentially detrimental pathogens originating from the upper and lower GI tracts. These regions serve as the primary abode for the vast majority of the microorganisms that comprise the body’s microbiota. These microorganisms are primarily found in the large and small intestines, as well as the oral cavity (111). The false conclusion was reached due to several factors: the inadequate achievement in isolating and cultivating gastric microbiota, the absence of rapid and non-invasive diagnostic tests, and the emergence of microarray and next-generation sequencing technology, which have focused on the bacterial 16S ribosomal RNA (16S rRNA) as the primary target for accurate taxonomy and phylogeny identifications (112).

### The prime pathogen: H. pylori

In 1982, Marshall and Warren discovered the significant revelation that *H. pylori* was the underlying factor responsible for both peptic ulcers and gastritis, thereby drawing attention to the possibility of stomach infection leading to cancer development (113). This particular microorganism, after recent discovery, has been categorized as a type I carcinogen and is projected to have an impact on over 50% of the global populace. In a tiny proportion of the afflicted population (2%), this infection results in a predictable step-by-step pattern of illness progression that, if discovered in time, can be reversed (114). The CagA protein, one of the cag pathogenicity islands, is a mechanism through which *H. pylori* infection can cause cancer (115, 116). Depending on the modifications after translation, CagA is initially introduced into the cell through the Type IV secretion system. Subsequently, it assumes a pathogenic role by stimulating the activation of SHP2, Abl, or Src kinases within the enclosure of GC (114). The EPIYA motif, which is distinguished by the existence of residues such as proline, isoleucine, glutamate, tyrosine, and alanine, functions as the site for phosphorylation within the CagA protein and may display discrepancies depending on the particular strain of *H. pylori* (117, 118). In addition, *H. pylori* can generate peptidoglycan within the cellular environment of the host, thereby augmenting the synthesis of IL-8 and cox, alongside other pro-inflammatory cytokines (119, 120). Consequently, this leads to the persistence of chronic inflammation and ultimately facilitates the emergence of cancer. It has been additionally established that *H. pylori* releases VacA toxin. This substance can potentially diminish T-cell responses and facilitate the formation of lesions with minimal opposition from the immune system (114, 121). Today, *H. pylori* may be detected via a quick urease test, a polymerase chain reaction test, a histological study of biopsy specimens, and a serological test. Infection with *H. pylori* typically develops in childhood and persists throughout the host’s life without antibiotic therapy. The transmission of bacteria can occur through direct contact between individuals, either through oral-oral or fecal-oral pathways (122). *H. pylori* is believed to persistently inhabit approximately half of the global populace, with approximately 15% of individuals afflicted by this pathogen subsequently progressing to the development of gastric ulcers (123). *H. pylori* employs flagella to facilitate its entry into the gastric mucosa, seeking refuge from the stomach’s highly acidic milieu. It is essential to acknowledge that a substantial percentage, surpassing 20%, of *H. pylori* variants adhere to the exterior of gastric epithelial cells (122). The ability of *H. pylori* to securely attach to the gastric epithelial cells is facilitated by the implementation of adhesion molecules, including the outer inflammatory protein A (OipA), sialic acid-binding adhesin (SabA), and adherence-associated lipoproteins (AlpA/B) (124).

### Dysbiosis of Non-*H. Pylori* microbiota in gastric cancer

For many years, *H. pylori* has been thought of as the predominant, if not the only, bacteria that can live in the stomach’s acid environment and encourage gastric carcinogenesis (111). However, new data from 16SrRNA sequencing showed that non- *H. pylori* strains co-occurred in both *H. pylori +* and *H. pylori* - persons with GC (125). Additionally, accumulating evidence indicates that the term “dysbiosis” best describes how the microbiome gradually changes throughout the development of GC (126, 127).

### Microbiota in prevention and therapy of GC: from mice to patients

Probiotics, traditionally limited to their use as food additives (such as in yogurt), have been revolutionized by advanced methods like fecal microbiota transplantation (FMT). These techniques have introduced the concept of therapeutically restoring eubiotics in GI illnesses, thereby transforming the field entirely (128). However, there hasn’t been any research done yet on the treatment or prevention of GC in humans. Notably, an international expert council has questioned FMT practices due to inconsistent results and the need for standardization and safety (129, 130).

### Probiotics: prevention of GC

Proton pump inhibitors (PPIs) are widely used, which has increased interest in how they may interact with the stomach flora. PPI use over an extended period decreases stomach acid output, encouraging bacterial proliferation because of increased pH brought on by *H. pylori*. PPIs thus have a significant influence on the variety and abundance of bacteria (131–133). For instance, significant levels of *Bifidobacteriaceae* from the oral cavity (*Bifidobacterium dentium*, *Scardovia inopinata*, and *Parascardovia denticolens*) were found in human stomachs with hypochlorhydria in gastritis patients on omeprazole (134, 135). PPIs also boost the number of organisms that may colonize the mouth, such as *Clostridiales*, *Streptophyta*, *Veillonella*, *Fusobacterium*, *Leptotrichia*, *Oribacterium*, *Porphyromonas*, *Prevotella*, *Capnocytophaga*, *Granulicatella*, *Campylobacter*, and *Bulleidia* (133, 136). Nonsteroidal anti-inflammatory drugs (NSAIDs) and PPIs can have adverse side effects on the GI mucosa, although taking probiotic strains along with them might lessen these effects. *Bifidobacterium*, as an exemplification, safeguards mice from the occurrence of stomach ulcers caused by aspirin, while *Lactobacillus plantarum*, derived from green tea, has curative properties against gastric ulcers induced by alcohol (137–139). In mice given the PPIs rabeprazole or vonoprazan, oral *Lactobacillus* johnsonii supplementation reduced indomethacin-induced minor intestine damage (140).

### Antibiotics: eradication of *H. pylori*

Large-scale clinical trials and field investigations have provided substantial evidence in favor of the cancer-preventive effects of *H. pylori* eradication. However, the emergence of antibiotic resistance poses a significant challenge, as does the disturbance of the gut microbiota and the impact of *H. pylori* on various other disease states, including asthma and esophageal cancer (141). Various microorganisms linked to stomach illness were found in a recent randomized controlled clinical investigation one year after *H. pylori* elimination (142). Probiotic Roseburia, *Faecalibacterium prausnitzii*, and *Sphingomonas* were decreased, whereas *Streptococcus anginosus*, *Acinetobacter lwoffii*, and Ralstonia were enriched. Also linked to chronic gastritis were oral bacteria (*Streptococcus*, *Peptostreptococcus*, *Prevotella*, *Rothia*, Parvimonas, *Granulicatella*). Other researchers who conducted endoscopic ablation of early GC in individuals experiencing a deficiency of beneficial microorganisms, such as *Ralstonia*, *Faecalibacterium*, *Blautia*, *Methylobacterium*, and Megamonas, observed a prolonged presence of dysbiosis in patients after the eradication of *H. pylori* (143, 144). The restoration of beneficial GI microbiota, such as *Bifidobacterium*, *Lactobacillus*, *Lachnoclostridium*, and *Blautia*, has been observed in young asymptomatic individuals following the eradication of *H. pylori*. Additionally, the presence of pathogenic Alistipes has been found to decrease (145, 146).

## The role of the microbiome in liver cancer

Liver cancer is the primary reason for cancer deaths, and its prevalence is rising yearly (147). About 90% of initial liver malignancies are hepatocellular carcinomas (HCC), a significant worldwide health issue (148). Several factors increase the risk of HCC, including chronic hepatitis B and C infections, alcoholism, metabolic liver disease (particularly nonalcoholic fatty liver disease), and exposure to food toxins like aristolochic acid and aflatoxins (149). The World Health Organisation predicts that by 2030, this illness will claim the lives of more than a million individuals (150). Sorafenib, an inhibitor of multiple kinases that has received approval for the management of hepatic carcinoma, occupies the position of primary therapeutic modality for advanced HCC. It has been shown to improve overall survival significantly, but it cannot stop the progression of the disease because of the emergence of resistance to antiproliferative therapies (151). The early detection and treatment of HCC contribute to the enhancement of its prognosis, which is also observed in the majority of disease processes. The most optimal opportunity to detect a medical condition at an early stage is by closely monitoring individuals with a heightened likelihood of developing the disease. This group includes both those who have cirrhosis of any kind and those who carry the hepatitis B virus (152). According to the 2012 NCCN recommendations, individuals at a heightened risk should undergo hepatic ultrasonography and AFP testing on a semiannual to annual basis. Per the 2012 recommendations of the National Comprehensive Cancer Network (NCCN), individuals classified as high-risk should undergo hepatic ultrasonography and AFP testing every six to twelve months (152, 153). The correlation between the presence of microorganisms causing infection and the onset of cancer has been recognized for a significant duration. Among the various mechanisms that contribute to this association, the chronic inflammation induced by infection is considered to be an important causative factor. Emerging evidence indicates that the resulting gut dysbiosis, characterized by an imbalanced state of intestinal microbial composition linked to illness, is responsible for the carcinogenic implications of these microbial stimuli. Consequently, this dysbiosis triggers chronic inflammation and, ultimately, the development of cancer (154). However, it is imperative to acknowledge that not all microorganisms are harmful. A plethora of commensal bacteria have a crucial function in fostering the development of the host’s immune system (155, 156). The host’s state of health is influenced by the constituent member types (pathogenic or commensal) and abundance arrangement (dysbiotic or eubiotic) of the intestinal microbial. Numerous investigations have effectively highlighted the crucial functions that gut bacteria play in the development of HCC (154, 157). The bidirectional interplay between the GI tract and the hepatic organ transpires via the portal vein, a conduit that expedites the passage of diverse entities originating from the gastrointestinal system, including nourishing compounds, metabolic byproducts of microorganisms, and constituents of said microorganisms, to the hepatic entity (158, 159). Once in the bile duct, these substances go from the liver to the gut. As a result of this enterohepatic circulation, the liver is constantly exposed to substances that originate from the gut (160). Furthermore, the association linking the gut and the liver, commonly called the “gut-liver axis,” has garnered increasing attention from researchers due to its pivotal role in preserving liver homeostasis and averting the onset of ailments (161, 162). One common finding in several liver illnesses is that tight connections between adjacent intestinal epithelial cells are impaired with increasing intestinal permeability, indicating that substances coming from the gut have an impact on liver function (160, 163). Furthermore, microbial dysbiosis in the lower GI tract and small intestinal bacterial overgrowth (SIBO) are linked to liver injury (164, 165). This finding implies that the bile produced by a healthy liver, along with other liver-derived compounds, contributes to the probiotic status of the gut microbiota (165). The liver is commonly perceived as an organ devoid of immunological function, instead playing a pivotal role in various metabolic processes, energy source storage, and detoxification (166, 167). The organ can also be perceived as a highly responsive component of the immune system, serving as a dwelling place for various immune cells such as Kupfer cells, natural killer (NK)/NKT cells, and T and B lymphocytes. Additionally, it harbors stromal cells such as liver sinusoidal endothelial cells (LSECs) and hepatic stellate cells (HSCs), which possess the ability to release cytokines and various other substances that can interact with immune cells (160).

### Hepatitis viral infection and gut microbiome

Hepatitis is a liver inflammation that can either go away independently or progress into a dangerous illness that causes cirrhosis or HCC. Hepatitis B and C virus infections typically result in chronic hepatitis, and viral infections are the primary cause of hepatitis worldwide (168). Both viruses cause host immune responses for clearance after infecting hepatocytes. To stop viral replication, nucleoside (or nucleotide) analogs (NAs) are frequently used to treat viral hepatitis. In addition, the mature gut microbiota is necessary for quick HBV clearance via efficient host immune boosting (154). The gut microbiota of individuals with cirrhosis caused by HBV exhibited notable distinctions compared to the gut microbiota of healthy control subjects, as indicated by a study conducted using advanced next-generation sequencing technology (169, 170). Specifically, certain bacterial species such as *Clostridium*, *Prevotella*, *Veillonella*, and *Streptococcus* displayed higher prevalence levels, whereas *Alistipes* and *Eubacterium* were found to be less frequently observed (171). The presence of oral microorganisms in higher quantities indicates that the transfer of microbes from the mouth to the gut is a prevailing occurrence among individuals with cirrhosis (171). The diversity of microorganisms in the GI tract, assessed using the Shannon and Simpson indices, declined in individuals with cirrhosis and recovered to a level comparable to that of healthy individuals in patients with HBV-related HCC (172, 173). In addition, the diversity decreased more in early hepatitis B patients than in intermediate cases, but not considerably, and both were lower than in healthy controls (174). Collectively, the variety of gut bacteria appears to decline during the early stages of HBV infection and then recover to a level comparable to that of healthy individuals as the liver disease advances. The aberrant bile acid production and composition, which compromises the bile acids’ antimicrobial defenses and enables the transfer of oral species, are thought to contribute to these gut microbial changes linked to HBV infection (154, 175).

### Microbiome and alcoholic liver disease

Alcohol liver disease (ALD), characterized by alcoholic hepatitis, alcoholic fatty liver disease, and alcoholic cirrhosis, represents a significant contributing factor to various liver ailments (176, 177). Alcohol’s metabolic byproducts are held responsible for the adverse effects of alcohol abuse. Alcohol undergoes oxidation within the hepatocyte, primarily resulting in the formation of acetaldehyde through the activity of alcohol dehydrogenase. Meanwhile, there is a limited production of reactive oxygen species (ROS). Prolonged exposure to acetaldehyde and ROS can lead to hepatotoxicity and carcinogenicity within the liver (178). Although the liver is the primary site of alcohol metabolism, intestinal enzymes and microorganisms are also capable of doing so (179, 180). Therefore, drinking too much alcohol increases luminal acetaldehyde and ROS, which disturbs the gut ecology by affecting gut barrier function and promoting gut dysbiosis, which alter the makeup of the gut’s microbial population (181–183). As substantiation, the presence of alcoholic hepatitis resulted in a decrease in *Akkermansia* in individuals with severe illness in comparison to individuals who were in good health, and this decrease was even more pronounced (184, 185). Mice that underwent an FMT from individuals suffering from acute hepatitis and alcoholism exhibited increased levels of *Bacteroides*, *Butyricimonas*, *Alistipes*, *Bilophila*, and *Clostridium* XIVa compared to mice that did not receive an FMT (186, 187). Additionally, the presence of alcoholic hepatitis resulted in an elevation of bacterial DNA levels in the bloodstream when compared to individuals who do not consume alcohol. This increase was characterized by a decrease in DNA from *Bacteroidetes* and an increase in DNA from *Fusobacteria* (188).

### Non-alcoholic fatty liver disease and microbiome

It is estimated that approximately 80-100 million individuals in the United States, constituting around 25% of the adult population, are believed to be affected by non-alcoholic fatty liver disease (NAFLD). The primary etiology of chronic hepatic ailment presently observed worldwide is NAFLD (189–191). Hepatic steatosis, a condition characterized by the accumulation of fat in the liver exceeding 5% of its overall weight, can be attributed to an excessive intake of alcohol. Abdominal imaging data suggests that the global prevalence of NAFLD is expected to reach 25%, with the African continent experiencing the lowest majority at 13.5% and the Middle East observing the highest at 31.8%. Non-alcoholic steatohepatitis (NASH) emerges in approximately 30% of individuals diagnosed with NAFLD (192). NASH can potentially progress from a state of simple steatosis to the more severe conditions of cirrhosis or HCC, or it may experience a decline in its condition (192, 193). It has been shown that NAFLD causes lipid metabolism to be dysregulated, resulting in the loss of CD4+ T cells and subsequently encouraging the development of hepatocarcinogenesis (194, 195). Similar to this, IgA+ cells that have accumulated in the livers of NASH patients with fibrosis help to promote hepatocarcinogenesis by inhibiting CD8+ T cell activation (196, 197). The importance of the intestinal metagenome in the etiopathogenesis of NAFLD has also been emphasized by recent findings (192). The initial step in establishing the etiological link between gut bacteria and NAFLD was replicating the hepatic changes associated with the disease in mice utilizing co-housing and FMT trials (198, 199). Dysbiosis, in turn, can lead to the development of metabolic disorders, including metabolic syndrome, obesity, Type 2 Diabetes Mellitus (T2DM), and NAFLD (200, 201). Increased *Enterobacteriaceae* was shown to be one of the characteristics that predicted NAFLD-cirrhosis, reflecting its significant involvement in the development of NAFLD (169). Two strains of *Enterobacteriaceae*, which belong to the *Klebsiella pneumonia* family, were fortuitously discovered to produce ethanol among Chinese individuals affected by NAFLD internally. This finding offers valuable knowledge regarding the development of NAFLD in individuals who do not consume alcohol (202). When compared to NASH cirrhosis, individuals with NAFLD-related HCC had lower levels of *Akkermansia* and *Bifidobacterium* species, indicating that gut dysbiosis may worsen the development of NAFLD to hepatocarcinogenesis (203, 204).

## The role of the microbiome in esophageal cancer

Esophageal cancer, acknowledged as a highly prevalent form of malignancy worldwide, is projected to have approximately 604,100 novel occurrences in 2020 (205). Nearly 80% of occurrences of this malignant tumor are found in less developed areas, which bear a disproportionately heavy burden. A discrepancy exists in the occurrence and mortality rates between males and females, with males representing approximately 70% of reported cases, resulting in a 2 to 5-fold difference (206). Moreover, the likelihood of developing esophageal cancer increases as individuals grow older, particularly in middle-aged and older demographics (207). In conjunction with the worldwide phenomenon of population growth and aging, the escalating prevalence of risk factors such as alcohol and tobacco consumption, inadequate dietary habits, sedentary lifestyles, and obesity is contributing to the rapid escalation of esophageal cancer globally (208, 209). esophageal cancer comes in two forms: esophageal squamous cell carcinoma (ESCC, also known as SCC) and esophageal adenocarcinoma (AC). AC is more typical in affluent nations, while ESCC is more widespread in East Asia, Southern Africa, East Africa, and Southern Europe (205). Based on the kind of cell from which cancer arises, SCC and AC exhibit significant differences in carcinogenesis (210). In terms of incidence during the previous forty years, AC has been shown to surpass SCC by a wide margin (108). SCC primarily impacts the upper and middle regions of the thoracic esophagus, arising from the squamous cells present within the mucosal lining of the esophagus. AC commences in the epithelial cells, most in the inferior thoracic esophagus (210). Although little is known about the esophageal microbiome, it is recognized that it is not a sterile portion of the digestive system (211). In the esophagus, food passes through quickly, likely limiting the prevalence of microorganisms. However, the pH in healthy people is very steady (about 7), which is ideal for various microbes. The esophagus is home to certain microbes, according to microbiome analysis (212, 213). It is worth mentioning that the composition of microorganisms inhabiting the lower, middle, and upper regions of the esophagus is indistinguishable (214). Most of the esophageal microbiome comprises six phyla, namely *Bacteroides*, *Firmicutes*, *Fusobacteria*, *Proteobacteria*, *Actinobacteria*, and TM7 (215, 216). There is a varied microbial community seen among the Gram-positive bacteria. Particularly, the esophagus of healthy people has the highest concentration of the Streptococcus genus (217). In addition, the esophagus also harbors *Prevotella* and *Veillonella* (211). The microbiome is changed by esophageal disorders. It is possible to identify esophageal illnesses by the unbalanced changes in the esophageal microbiome (218, 219). Recent research has expanded our understanding of the connection between changes in the gut microbiota and the development of esophageal cancer. It has been proposed that this connection may be essential for the creation and growth of tumors (220). Blackett et al. (221) showed that individuals with Barrett’s esophagus and gastroesophageal reflux disease (GERD) had significantly higher concentrations of *Campylobacter*. It is believed that *campylobacter* causes the esophageal mucosa to become inflamed, followed by epithelial metaplasia, which finally results in malignant transformation (222). Elliott and colleagues (223) discovered that certain strains of *Lactobacillus* are concentrated within tumors in roughly 50% of AC patients and that microbial diversity diminishes in AC while relative *Lactobacillus fermentum* abundance rises. Zaidi et al. (224) found that AC contains large amounts of *E. coli*. Additionally, there was a significant increase in the expression of several Toll-like receptors (TLR1, 3, 6, 7, and 9) within the neoplastic tissue of a rat model mimicking AC. Etiological investigations have elucidated that *H. pylori* has the potential to mitigate the occurrence of AC through the suppression of gastric acid secretion, thereby reducing the likelihood of reflux esophagitis while also modulating the quantity of T cells (225). *H. pylori*, on the other hand, has been shown to cause GERD to manifest. Several studies have established a correlation between *Tannerella forsythia* and an increased likelihood of AC. Conversely, symbiotic *Streptococcus pneumoniae* and *Neisseria* have been associated with a decreased risk of AC. Notably, the enrichment of *Porphyromonas gingivalis* (*P. gingivalis*) has been identified as a significant risk factor for ESCC, as highlighted by various investigations (226–230). *P. gingivalis* induces the process of epithelial-mesenchymal transformation (EMT) using the transforming-growth-factor (TGF)-dependent Smad/YAP/TAZ signaling pathway, and also triggers the activation of the nuclear factor (NF)-B signaling pathway, thereby stimulating the proliferation and metastasis of ESCC cells (231, 232). It has been proven that the microbiome of the esophagus contains viral DNA from the Gammapapillomavirus, Betaherpesvirus, and Gammaherpesvirus. With the discovery of Papillomavirus (HPV) DNA from esophageal neoplasia, the probability of developing ESCC was increased in the presence of EBV and HPV infections (233). Fungi infections with inflammation are common in esophageal cancer patients, which may suggest a possible link with the development of esophageal cancer (233). In research by Deng et al. (234) comprising 23 esophageal cancer patients and 23 matched healthy persons, the gut microbiome was examined. By 16S rRNA gene sequencing, the gut microbiota was examined from fresh stool samples. When the strain was considered, it was shown that esophageal cancer patients had much larger amounts of *Actinobacteria* and *Firmicutes* and lower levels of *Bacteroidetes* than healthy people. According to scientists, individuals with esophageal cancer had lower levels of bacteria that produce SCFAs while having higher levels of bacteria that produce lipopolysaccharides (LPS) (234, 235). The significance of the pool of SCFAs ought to be underscored as it possesses various benefits, including its ability to mitigate inflammation and enhance the structural integrity of the intestinal barrier. It is of utmost importance to acknowledge that anaerobic microorganisms located in the distal regions of the GI tract synthesize butyrate, thereby suggesting that it may exert a substantial influence on the pathogenesis of neoplastic growths within this system, encompassing esophageal carcinoma (236). In a study conducted on patients with ESCC, it was discovered that the presence of *Fusobacteria*, *Bacteroidetes*, and *Spirochaetes* was notably reduced (n=18) (176). The high-fat diets (HFD) negatively impact the bile acids composition and the gut flora. According to research in mice, the modifications in bile acid composition brought on by HFD may aid in the emergence of Barrett’s esophagus and esophageal cancer (205).

## Non-bacteria microbiome (virus, fungi, and archaea) in GI cancer

**Figure 2:**
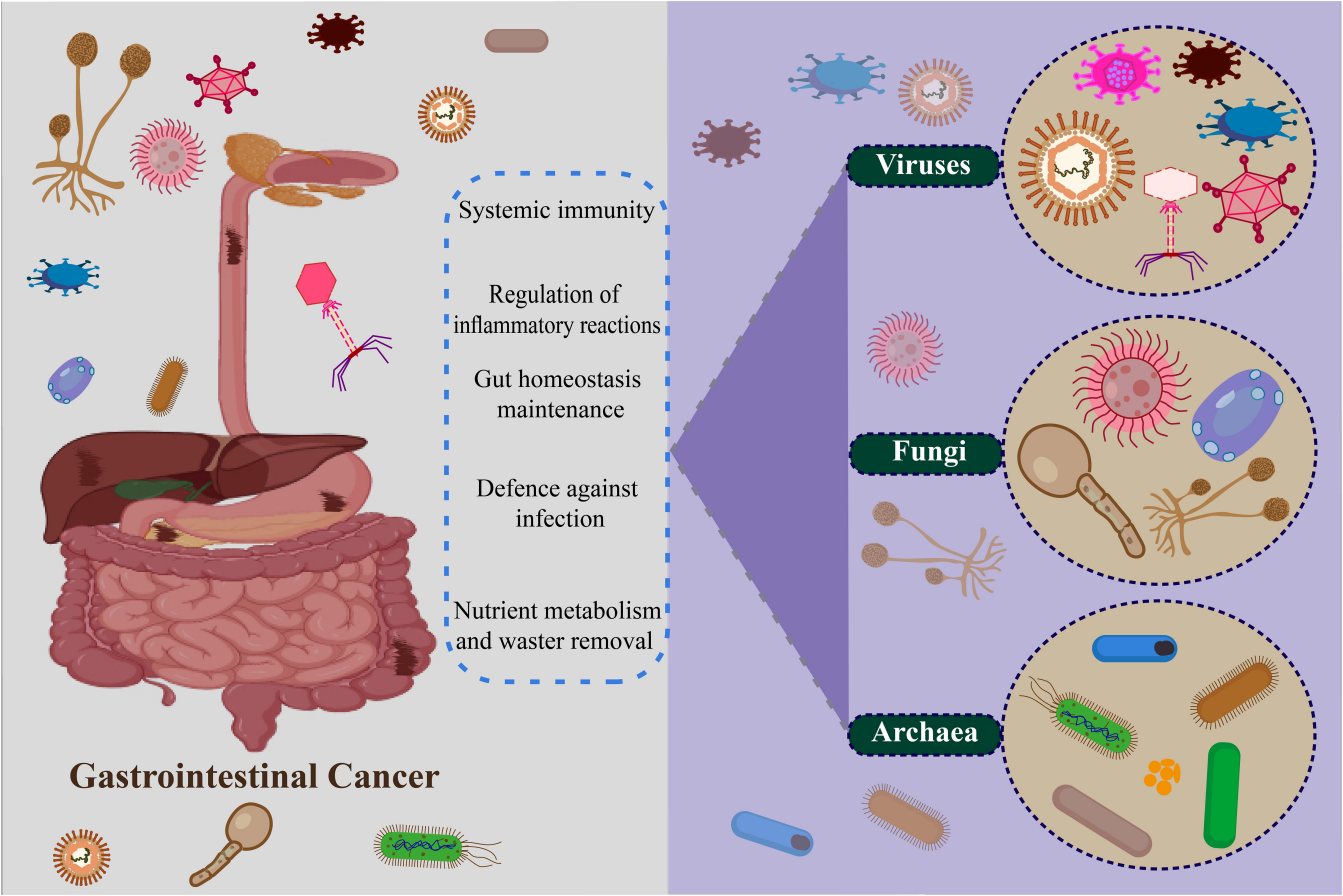
Non-bacteria microbiome (virus, fungi, and archaea) in gastrointestinal cancer.

Bacteria are the predominant microorganisms found throughout the GI tract (237). The impact of specific species or the combined bacteriome on GI cancers has been extensively researched (238). However, in recent times, the presence of viruses, fungi, archaea, and microscopic eukaryotes in the GI tract has been confirmed due to the progress made in sequencing technology and biotechnology (233) (**Figure 2**).

### Viruses in GI cancers

Viruses exhibit a comparatively reduced presence in the gut when compared to bacteria, yet they have been identified as constituents of the enduring commensal microbial consortium within the GI tract (230, 239–241). Viruses have a notable impact on GI cancers. The human virome, encompassing the entirety of viruses found within the human body, is a vital component of the human microbiota and aids in the preservation of tissue equilibrium (242). Bacteriophages have been predominantly recognized within the microbiome, where they are ascribed to various functions. The functions encompassed within this domain encompass the regulation of bacterial populations through the cyclic processes of phages, namely lysogenic and lytic. The proportions of lytic and lysogenic phages are said to have a relationship with the bacteriome and are linked to the overall health condition of an individual (241, 243). Lysogenic bacteriophages might also play a role in the targeted establishment of bacteria and improving the fitness of host bacteria through the exchange of genetic material within the GI tract (243, 244). The involvement of viruses in the development of GI cancers is evident as they impact the abundance of these viruses, infect the cells of the epithelium, or alter the composition of the bacterium (245). A multi-cohort study was conducted in which fecal samples were analyzed using shotgun metagenomics to investigate the virome shift in patients with CRC compared to healthy individuals. Additional examination revealed that there was a fluctuation observed in the colon bacteriophages, with variations evident in both the early and late stages of CRC (246, 247). The analysis conducted subsequently revealed that there was a variation in the displacement of the colon bacteriophages between the initial and advanced stages of CRC (248, 249). *Epsilon15likevirus*, *Betabaculus virus*, *Punalikevirus*, and *Mulikevirus* exhibited a noteworthy augmentation in CRC individuals, thereby being linked to escalated intensity and fatality rates. It has been suggested that viruses belonging to the eukaryotic colon could potentially disrupt the balance of the immune system and trigger modifications in the DNA via mechanisms that are dependent on the presence of the virus (250). Indeed, there is a growing body of evidence suggesting that infections caused by eukaryotic viruses are linked to an elevated risk of CRC (251). In cancerous tissues of CRC patients, there was a presence of viral infections including HPV, human herpesviruses, human polyomaviruses, human bocavirus, and Inoue-Melnick virus in comparison to the surrounding normal tissues (252, 253). In the same manner that tumor tissues of CRC patients exhibited the presence of viral DNAs, similar findings were observed in the GC tissues. The well-documented role of Epstein-Barr virus (EBV) as an etiological agent in the development of GC further supports this observation. EBV-positive gastric carcinoma is distinguished by distinct genomic abnormalities and clinicopathological characteristics (254, 255). After being infected, the EBV incorporates its DNA into the host organism. Subsequently, it manifests latent protein and disrupts DNA methylation through the influence of miRNA under the presence of the latent protein. This process ultimately leads to the development of EBV-positive GC (255, 256). The prevalence of human cytomegalovirus (HCMV) is extensive within human populations, encompassing a range of infectious microorganisms. HCMV has been documented to endure within the host for extended durations after the initial infection (257). Recent research has placed greater emphasis on the connections between HCMV and several types of malignancies, such as glioblastoma, breast cancer, GC, and CRC (258). HCMV was observed to exhibit a greater presence in GC tissue compared to the surrounding normal tissues, thus suggesting its potential involvement in the development of carcinogenesis and potentially facilitating the lymphatic metastasis process in GC (259). Furthermore, HCMV has also been documented to elicit disturbances in the GI tract, such as inflammatory bowel disease, ulceration, erosion of the cell wall, and hemorrhage in the mucosal lining (258). Reports have also been documented regarding the identification of viruses, particularly bacteriophages, within the esophageal microbiome. DNA viruses including betaherpesvirus, gammaherpesvirus, and gammapapillomavirus were also found (20, 260). Due to their primary focus on bacteria, it is conceivable that the correlation between the virome of the esophagus and adenocarcinoma could be elucidated in investigations involving larger groups of subjects. In addition, it has been reported that infections caused by EBV and HPV are associated with a heightened susceptibility to ESCC (261). Latent gammaherpesvirus 68 infection in a mouse model exhibited the capacity to induce persistent immune system stimulation, thereby safeguarding against pathogenic infection caused by *Listeria monocytogenes* (262, 263).

### Fungi in the GI cancer

Fungi have established their presence as inhabitants of the GI tract of individuals in good health, although their composition is primarily influenced by lifestyle factors, particularly dietary choices (264). These microorganisms have been observed to exist within the gastric compartment, colon, pancreas, and esophagus, albeit in a significantly smaller ratio when compared to bacteria (265, 266). Recent research is commencing to unveil the significance of fungi in the GI tract. A variety of fungi such as *Candida*, *Cryptococcus*, *Saccharomyces*, *Malassezia*, *Debaryomyces*, *Cladosporium*, *Trichosporon*, and *Galactomyces* have been documented as inhabiting the gastrointestinal tract of healthy individuals (264). Fungi play a crucial role in sustaining the equilibrium of the GI tract. Furthermore, they have been demonstrated to possess functions in systemic immunity, regulation of inflammatory reactions, and protection against infectious agents (267). Fungi are purported to stimulate the activation of T helper 17 cells, which play a crucial role in safeguarding the host against infections. Moreover, these cells contribute to the development of secondary lymphoid organs and the fine-tuning of the host’s immune and inflammatory responses (268, 269). Fungal species, namely *Candida albicans*, *Saccharomyces cerevisiae*, and *Candida glabrata*, have been detected in the esophagus of individuals in a non-pathogenic state (270). Candida and Phialemonium demonstrate the capacity to endure the harsh acidic conditions prevalent within the ecosystem, specifically within the gastric fluids (271, 272). The ratio between Basidiomycota and Ascomycota was found to be imbalanced in patients with CRC, similar to other diseases affecting the intestines. In individuals with CRC, an elevation in the population of *Malasseziomycetes* fungi and a decrease in the abundance of *Saccharomycetes* fungi were noted (273, 274). The composition of fungal genera including *Aspergillus*, *Rhodotorula*, *Kwoniella*, *Pseudogymnoascus*, *Malassezia*, *Talaromyces*, *Moniliophthora*, *Debaryomyces*, *Pneumocystis*, and *Nosemia* experienced changes in CRC cases, a finding that was confirmed in separate cohorts of Chinese and European populations (275). In an experimental mouse model investigating esophageal cancer, the administration of oral fungi *Cladosporium cladosporiodes* was found to enhance the severity of ESCC. Interestingly, this detrimental effect was effectively counteracted by treatment with antifungal agents (276). Additionally, infections caused by the *C. albicans* fungus were documented in patients suffering from ESCC (277). The presence of an imbalance in gastric fungi was observed in individuals with GC. The profile of the fungal community (mycobiome) in GC patients exhibited notable differences, including a decrease in diversity, compared to the control group. *Candida* and *Alternaria* exhibited an increased concentration in the GC, whereas *Thermomyces* and *Saitozyma* experienced a decrease in abundance within the GC (278, 279). Similar to other GI microbiome members, alterations in the resident mycobiome that impair their functioning, manipulation of the whole microbiome, or infection by specific pathogenic fungus species may all influence GI malignancies (280, 281).

### Archaea in GI cancer

The progress in the field of sequencing and the analytical methods used in bioinformatics have facilitated the examination of archaea, a group that has received less attention in comparison to bacteria, viruses, and fungi within the intestinal ecosystem (282). Archaea represent a distinctive assemblage of prokaryotic organisms characterized by their lack of D-glycerol, esters, fatty acids, and peptidoglycan (283, 284). Owing to their cellular composition, these organisms were recognized for their ability to colonize harsh habitats such as those characterized by high temperatures, alkaline conditions, acidic conditions, and high salinity levels (285). Archaea were commonly presumed to inhabit environments characterized by severe ecological conditions, nevertheless, recent investigations have ascertained their presence in mesophilic conditions comprising human skin, oral cavity, nasal passages, vaginal region, and the GI tract (286). In the GI tract, there have been documented occurrences of both methanogenic archaea and haloarchaea, with their respective proportions being subject to variation dependent on the individual (287). Methanogenic archaea facilitate the reduction of carbon dioxide through the process of methanogenesis, occurring in the GI tract during nutrient digestion. This metabolic activity effectively assists in the elimination of hydrogen from the gut (288, 289). Colonic archaea have also been found to play a role in the elimination of trimethylamine (TMA) from the GI tract. TMA is generated as a byproduct during the degradation of choline mediated by colon microorganisms, and its presence has been linked to elevated probabilities of atherogenesis and the development of cardiovascular ailments (290, 291). The activation of antigen-specific adaptive immune responses by Archaea is a phenomenon that should not be overlooked, as it has the potential to play a crucial role in maintaining immune homeostasis within the GI tract (292). Distinct groups of archaea in the colon were observed in individuals with CRC and colorectal adenoma in comparison to those who were in good health, thus suggesting a modification during the various phases of the development of cancer (293). The presence of Archaea has also been linked to the emergence of IBD, anorexia, and anaerobic abscesses (294, 295) (**Table 1**).

**Table 1:** A summary of studies relating the gut microbiome to GI cancers.

Type of cancer	Methods Used	Conclusion	References
**CRC**	The study analyzed the microbial communities in the colon and the genetic variability of Fusobacterium in 43 Vietnamese patients with CRC and 25 individuals with non-cancerous colorectal polyps. This was achieved through the utilization of 16S rRNA gene profiling, anaerobic microbiology, and comprehensive genome analysis	- *F. nucleatum* consistently demonstrates an association with CRC. - The diagnostic and therapeutic options can utilize the genomic diversity present in *Fusobacterium*.	Tran et al. (296)
	The analysis of 18 surgical specimens of human CRC was conducted using 16S rRNA gene sequencing. - Differential examination of microbiomes in tissues and mucus	*-Enterobacteriaceae* and *Sutterella* exhibit a higher presence in the mucus layer that envelops the mucosa. - *Rikenellaceae* exhibits a higher concentration within the mucosal layer that overlays cancerous tissues.	Tajima et al. (297)
	-Using a reverse microbiomics (RM) strategy. -Comparative genomics analysis using Vaxign	- The utilization of the RM methodology was implemented in order to predict the presence of 18 autoantigens and 76 potential virulence factors. - Proposed new model of CRC pathogenesis involving riboflavin synthase	Wang et al. (298)
	- Culture-independent methods for identifying bacterial populations - Sequencing V1-V3 or V3-V5 variable regions of bacterial 16S ribosomal RNA	- The presence of probiotic strains has the potential to impact the treatment of CRC. - Additional investigation is required to ascertain the most effective treatment.	Kim et al. (299)
	N/A	- The intestinal microbiota plays a crucial role in the advancement of colorectal cancer. - The potential of the intestinal microbiota to function as a biomarker in the prompt identification of CRC is considerable.	Ren et al. (300)
	- A systematic search find clinical studies published in the last two decades. - A comprehensive analysis was conducted on the following subjects: dietary interventions, potential biomarkers for CRC, probiotic administration in non-surgical patients, and probiotic administration in surgical patients.	-Bacterial metabolism exhibits a robust correlation with the development of colonic carcinogenesis and is subject to dietary influences. - Probiotics and prebiotics function as agents that can modify the microbiota by inhibiting the proliferation of epithelial cells and counteracting DNA damage. - As supplementary treatments to surgery or chemotherapy, *Bifidobacteria* and *Lactobacilli* reduce complications.	Fratila et al. (301)
	- A comprehensive search of the literature was conducted on March 3rd and 4th, 2023.	-Research indicates that biomarkers based on oral microbiota show potential as a non-invasive means of identifying CRC. However, additional studies are required in order to comprehend the mechanisms of oral dysbiosis.	Negrut et al. (302)
	- To facilitate the screening process, pertinent articles were extracted from different databases by utilizing specific keywords and phrases.	-CRC is linked to imbalances in the GI microbiome.	Eastmond et al. (303)
	- A systematic review of 2009. - Patients diagnosed with any stage of CRC were enrolled in the study.	-Microbiome composition could potentially influence the outcomes of surgery for CRC, although the available evidence is currently limited.	Lauka et al. (304)
	- A Mendelian randomization (MR) study was conducted using a two-sample approach in order to elucidate the causal relationship between the CRC and gut microbiota. - A thorough examination was performed on a total of 166 bacterial characteristics spanning four hierarchical levels: species, genus, family, and order.	- The inquiry confirmed the causal correlation between the gut microbiota and CRC, positing a possible linkage between genes and pathogenic microbiota in CRC. - The examination of the GI microbiome and its comprehensive analysis involving multiple omics techniques are of utmost importance in the endeavor to impede and manage CRC.	Xiang et al. (305)
	- The study evaluated the effectiveness of microbiome-derived biomarkers using noninvasive samples. - A study of 28 studies found that only two explored the co-metabolome as a potential biomarker for colorectal cancer and advanced adenoma patients.	- Based on the current evidence, it is not yet appropriate for routine clinical implementation to utilize the potential of the fecal and oral gut microbiome in order to improve CRC screening tools.	Zwezerijnen et al. (306)
	-A meta-analysis of fecal metagenomics sequencing data from 11 studies involving 692 patients with CRC and 602 healthy controls evaluated features associated with CRC.	- In this investigation, significant correlations were found between CRC status and colibactin, fadA, and *F. nucleatum* compared to control subjects. - Several distinct microbial species were found to be selectively enriched in young patients diagnosed with CRC.	Kharofa et al. (307)
**Gastric**	- The cutoff value for *H. pylori* infection is determined using pyrosequencing. - Extragastric microbiome is investigated using animal models.	- Relationship between GC and gastric microbiome. - There has been limited advancement in comprehending the non-*H. pylori* function.	Yang et al. (308)
	- Five patients were diagnosed with GC, non-atrophic gastritis, and intestinal metaplasia of the intestinal type.	-The diversity of bacteria declined progressively from non-atrophic gastritis to intestinal metaplasia to GC. - There was a noticeable disparity in microbiota between non-atrophic gastritis and GC	Aviles et al. (309)
	- New methods for identifying microbes in the stomach using molecular techniques have been developed. - Studies conducted on the INS-GAS transgenic mouse model	- The development of GC is influenced by the presence of gastric microbiota. - Microorganisms are linked to individuals diagnosed with GC.	Stewart et al. (310)
	N/A	- The significance of the gastric microbiome in the development of cancer is not substantial. - *H. pylori* and inflammation play significant roles in the development of GC.	Engstrand et al. (311)
	- Nucleotide sequencing techniques - Biocomputational tools	- Chronic inflammation is linked to GC. - *H. pylori* and other bacteria contribute to the development of GC.	Schulz et al. (312)
	-The study consisted of 48 individuals diagnosed with GC and 120 individuals without cancer. This group comprised of 20 individuals with normal gastric mucosa, 40 individuals with atrophy, 20 individuals with gastritis, and 40 individuals with intestinal metaplasia	- The group that was under control exhibited the most significant overall bacterial alpha diversity measurements, with the groups with intestinal metaplasia and cancer following closely behind. - The groups with atrophy and gastritis exhibited the lowest level of diversity.	Gantuya et al. (313)
	-The study included 60 individuals diagnosed with chronic gastritis, 30 individuals with early GC, and 30 individuals with advanced GC.	- The inquiry revealed significant variations in the microbial profile and composition when contrasting the initial and progressed stages of GC.	Wang et al. (314)
	- A total of 1630 individuals who were infected with asymptomatic *H. pylori*. -The individuals assigned to undergo *H. pylori* eradication therapy numbered 817, whereas the placebo group consisted of 813 individuals	- The elimination of *H. pylori* has the potential to offer extended defense against GC in populations at high risk, especially for those individuals who are initially infected with the bacteria but do not possess precancerous gastric lesions.	Yan et al. (315)
**Liver**	- Characterization of intestinal microbial composition in mice and humans - Using bacteriotherapy and antibiotics as potential therapeutic choices is being explored.	- The impact of modifications in the gut microbiota on the progression of hepatic malignancy is of considerable importance. - Bacteriotherapy possesses the capacity to modify the composition of microbiota and decrease inflammation.	Moreno et al. (316)
	- This review analyzes existing evidence and examines potential mechanisms. - Possible therapeutic applications are being discussed	- The microbiota of the GI tract contributes to the development of liver cancer. - Potential therapeutic uses consist of probiotics and FMT.	Zhou et al. (317)
	- Between mice that were free from germs and mice that were. - Alternatives such as gnotobiotic or humanized models were employed.	- The dysbiosis of the GI microbiota exerts a substantial influence on the progression of hepatic disorders. - Gnotobiotic models are applicable for microbiome research.	Hartmann et al. (318)
	N/A	- The connection between an imbalance in liver diseases and gut microbiota. - Therapeutic strategies may be developed by manipulating microbiota.	Abe et al. (319)
**Esophageal**	N/A	- Microbiota diversity and uniformity decline in cases of esophageal cancer. - The prevalence of Gram-negative bacteria is elevated in esophageal cancer.	Moreira et al. (320)
	- Comparative metagenomic approaches - Sequencing of the 16S rRNA gene	- The imbalance of the microbiota can lead to esophageal tumorigenesis. - The identification of microbiota has the potential to enhance the methods of EC treatment.	Zhou et al. (220)
	- Analysis of bacteria at genus level in gut - Principal coordinate analysis (PCoA) and analysis of similarities (ANSOIM)	- The gut microbiota could potentially play a role in the pathogenesis and progression of esophageal squamous cell carcinoma. - Certain gut bacteria may serve as biomarkers for the screening of these types of cancer.	Shen et al. (321)
	Next-generation sequencing techniques.	- *Streptococcus* is the predominant bacterial group found in the normal esophagus. - Gram-negative bacteria are more prevalent in the diseased esophagus.	Park et al. (322)
	- 16S rRNA gene sequencing - Bioinformatics analysis	- ESCC patients exhibit unique microbial features in comparison to individuals who are in good health. - The development of ESCC may be influenced by the microbiome present in the esophagus.	Lv et al. (323)

N/A, No Answer.

## Microbiomes and therapies for gastrointestinal cancers

### Chemotherapy

It is widely established that systemic chemotherapies impact both healthy GI tract cells and cancer cells. The microbiome will undoubtedly experience a disturbance, thus resulting in dysbiosis, which refers to an interruption in the typical makeup of the microbiome. Chemotherapy has been demonstrated to possess a wide-ranging impact on the microbiota, leading to a reduction in the variety of microorganisms. This reduction is accompanied by an elevation in the abundance of Firmicutes and a decline in Bacteroidetes (324). Moreover, gram-negative bacteria tend to increase while gram-positive bacteria decrease due to chemotherapy treatment. Even though various chemotherapy regimens may have distinct effects on the composition of the GI microbiota, this assertion remains valid (324, 325). The gut microbiota controls toxicity, anticancer effects, and medication effectiveness to control host reactions to chemotherapy medicines (326). The TIMER mechanistic paradigm presents an opportunity to alter the connection between the GI microbiota and chemotherapeutic medications using immunomodulation, translocation, enzyme degradation, metabolism, and ecological variation (327). Yamamura and colleagues (328) have discovered a significant association between the intratumoral DNA of *F. nucleatum* and the response of patients’ ESCC to neoadjuvant treatment. Liu et al. (384) have found that through autophagy, the intracellular bacterium *F. nucleatum* provides chemoresistance to ESCC cells. By inducing the activation of autophagy and enhancing the expression of autophagy-associated genes, *F. nucleatum* specifically acts upon the TLR4/MyD88 signaling pathway, leading to a reduction in the levels of miRNA-4802 and miRNA-18a. Consequently, this molecular modulation results in developing resistance to chemotherapy in CRC. In patients with CRC who are undergoing adjuvant chemotherapy, it has been observed that the presence of *F. nucleatum* infection leads to a reduction in the effectiveness of 5-Fluorouracil (5-FU) treatment by regulating the baculoviral IAP repeat containing 3 (BIRC3) through the TLR4/NF-B signaling pathway (329, 385). In a mouse CRC model, *Mycoplasm hyorhinis* may metabolize gemcitabine into inactive 2’, 2’-difluoro deoxyuridine via the CDDL gene (330). Furthermore, it has been established that the majority of the bacteria in PDAC are *Gammaproteobacteria*, which possess the CDDL gene necessary for the metabolization of gemcitabine. Ciprofloxacin can counteract this impact (331). Different commensal microbiota can alter the tumor microenvironment, impacting how well conventional chemotherapy works. By lowering the generation of ROS, the lack of *Lactobacillus* reduces the cytotoxicity of oxaliplatin (332). In one study, Chinese patients receiving FOLFOX treatment for low-lying rectal tumors had their gut microbiomes examined by fecal samples. It has been demonstrated that FOLFOX reduces the variety of the whole microbiome, and intriguingly, this diversity was reduced in patients who reacted to the FOLFOX rather than in nonresponders (333–335). *Lactobacillus rhamnosus*, a probiotic, improved the effectiveness of capecitabine against mouse GC growth (336). Cyclophosphamide (CTX) inhibits several immunological signaling cascades to produce its anticancer action (337). Preclinical research has revealed that some bacterial species, such as *Enterococcus hirae (E. hirae)*, are necessary for CTX-induced immunological activation. To activate the host immune response, CTX causes the bacteria to relocate to the spleen and lymph nodes. The anti-tumor action of CTX is likewise dependent on *E. hirae* and *Barnesiella intestinihominis*, according to further investigations (338, 339). By controlling antitumor cytotoxic CD8+ T cell responses and stimulating the IL-12 signaling pathway, butyrate may increase the effectiveness of oxaliplatin (340). According to prospective research, individuals with locally advanced rectal cancer (LARC) may benefit from using the gut microbiota as possible biomarkers to gauge how well they respond to neoadjuvant chemotherapy and radiation (341).

### Immunotherapy

**Figure 3:**
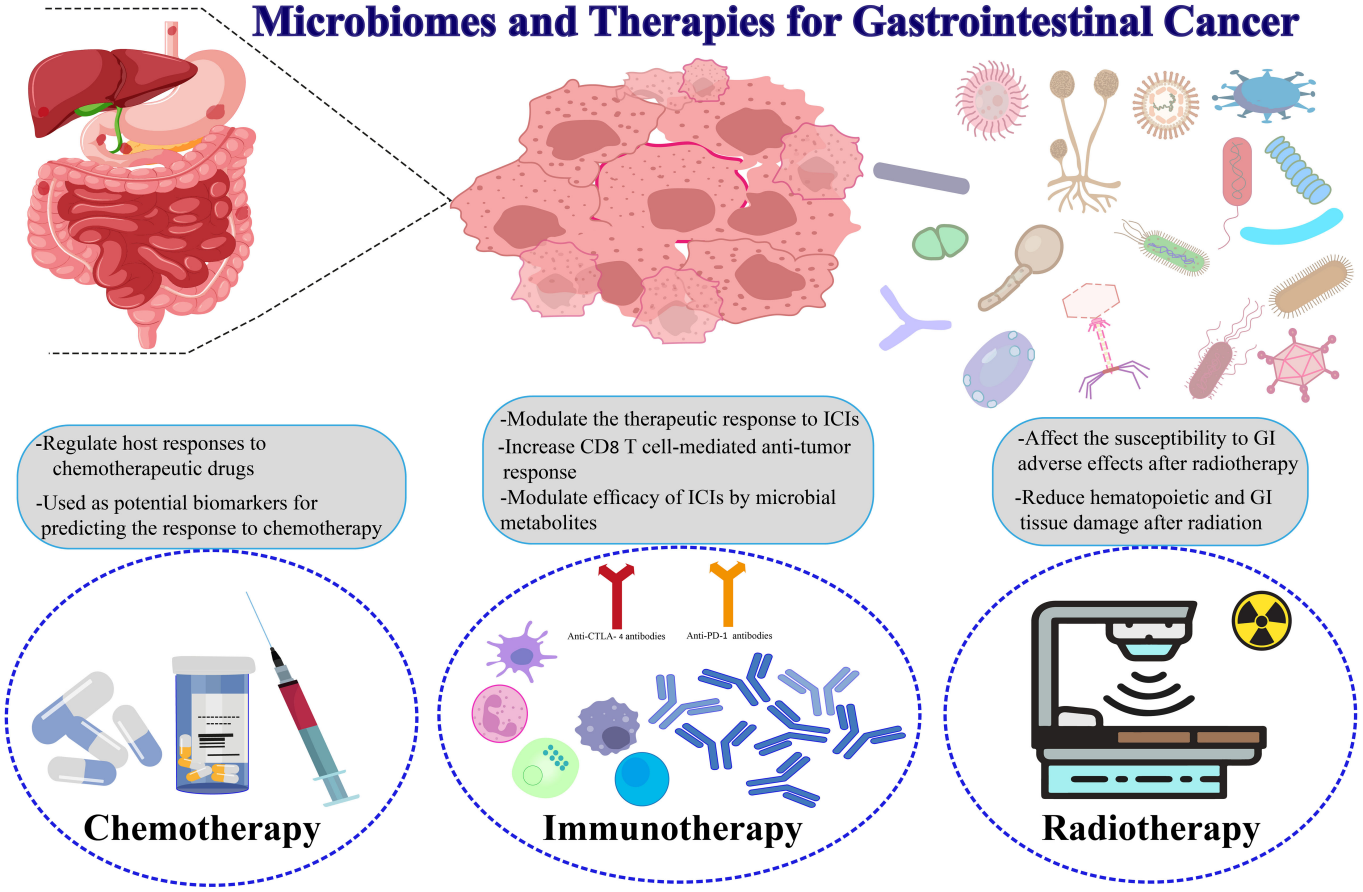
Microbiomes and therapies for gastrointestinal cancers.

The identification of immunotherapy, a therapeutic approach that harnesses the immune system of the body to tackle cancer, has emerged as a highly promising domain in the realm of cancer therapy (342). In the treatment of GI cancers, especially those that exhibit microsatellite instability (MSI-H), the utilization of immune checkpoint inhibitors (ICIs) like anticytotoxic T lymphocyte-associated protein 4 (CTLA-4) and anti-programmed cell death 1 (PD1) antibodies is being implemented (343–345). While dysbiosis can establish a connection between the microbiome and carcinogenesis, it is also plausible that a microbiome in good health possesses substantial potential to bolster antitumor immunity (346, 347). It has been proven that the therapeutic agents pembrolizumab and nivolumab, which function as inhibitors of PD-1, exhibit enhanced clinical efficacy in the prevalence of *Akkermansia muciniphila* and *Bifidobacterium* (348, 349). *B. fragilis* and *Bacteroides thetaiotaomicron* are also linked to the effectiveness of anti-CTLA-4 antibodies like ipilimumab (350). As the FMT from individuals who responded to ICI and those who did not into mice was carried out, it is noteworthy to observe that the microbiome of ICI responders exhibited a sustained augmentation of the anti-PD1 effects compared to the nonresponders. This observation highlights the intrinsic capability of the microbiome to stimulate the immune response against tumors (351). The interactions between ICI and microbiome highlight the crucial role that the host microbiome and tumor microenvironment may have in forecasting the response to treatment (351, 352). The potential impact of the microbiome on the effectiveness of ICIs suggests that it could also have a substantial role in regulating immune-related adverse events (iRAEs) associated with ICIs (353, 354). In the context of a practical inquiry, individuals afflicted with severe iRAEs exhibited heightened incidences of *Streptococcus*, *Faecalibacterium*, and *Stenotrophomonas* (355, 356). The concept of harnessing the GI microbiota to enhance the production of the anti-inflammatory compound known as butyrate by the gut microbiota to prevent colitis induced by ICI has already been discussed (357). The utilization of antibiotics to eliminate microbiota appears to diminish the efficacy of immunotherapy (358). In fibrosarcoma, melanoma, and CRC mice models, a combination of ampicillin, colistin, and streptomycin was demonstrated to impede the inhibition of CTLA-4 and subsequently revive the growth of tumors (359). A recent investigation discovered that in mice subjected to anti-CTLA-4 treatment, the administration of Bifidobacterium potentially diminishes autoimmune adversities. However, the absence of vancomycin exacerbates immunotherapy-induced colitis (360). The significant microorganisms that serve as predictive biomarkers for immunotherapy response were identified thanks to these studies. A study conducted on a rat colon adenocarcinoma model has identified a group of 11 bacterial strains that could potentially enhance the efficacy of immunotherapy (361). It’s interesting to note that probiotics have been examined as adjuvants in cancer therapies. In a murine model of CRC, the administration of cell lysates derived from Lactobacillus acidophilus in conjunction with a monoclonal antibody targeting CTLA-4 induced a substantial augmentation in CD8+ T lymphocytes, specifically the effector memory subset, along with a noteworthy reduction in regulatory T cells (Tregs). Additionally, the synergistic combination recovered animals with CRC-induced dysbiosis and reduced the aberrant abundance of *Proteobacteria* in the tumor microenvironment (362). Therefore, using immunotherapy in concert with probiotics may considerably aid the development of innovative therapeutic methods against CRC (363, 364) (**Figure 3**).

### Radiotherapy

Uncertainty persists over how gut microbiota controls the effectiveness of radiotherapy. Radiotherapy can augment the overall immune response regulated by the immune system in addition to the cytotoxicity of tumors (365) (**Figure 3**). In addition to causing tumor cell death, local irradiation can boost systemic immunity and inflammation. The therapeutic utility, however, was limited due to adverse outcomes, including bystander effects on adjacent cells, genomic instability, and alterations to commensal microorganisms (366). Research indicates that the microbiota residing in the GI tract could potentially exert a notable influence on the efficacy of radiation therapy (367, 368). The inhibition of apoptosis in cancer cells and the prevention of local immunocyte infiltration were observed when comparing germ-free mice to conventional mice with radiation. The implications of these findings suggest that the commensal microbiota could potentially have a positive impact on the regulation of the body’s reaction to radiotherapy treatment (369, 370). In experimental mice and humans getting radiotherapy, the gut flora is destroyed, which may lead to colitis and diarrhea partially mediated by IL-1β (371). Intestinal cell apoptosis and intestinal barrier function degradation are further side effects of radiotherapy that might result in intestinal inflammation (372). Further investigation revealed that angiopoietin-like 4 (ANGPTL4), a protein lipoprotein lipase inhibitor, plays a crucial role in radiotherapy damage resistance (373). *Streptococcus*, *Lactobacillus*, and *Bifidobacterium spp* stimulated the expression of ANGPTL4 to shield germ-free mice and regular mice from the harmful effects of irradiation (369). Additionally, butyrate, a widely recognized advantageous microbial byproduct, was demonstrated to enhance the efficacy of radiation in preclinical patient-derived CRC organoid models, suggesting the potential utilization of butyrate in combination with other therapies for cancer management (374). Additionally, a clinical investigation showed that formulations including *Lactobacillus casei*, *L. acidophilus*, and *B. bifidum* might reduce the intestinal adverse effects of radiation exposure (375, 376). *Lactobacillus rhamnosus*, although it possesses the ability to facilitate the recovery of radiation-induced damage to the intestinal mucosa, induce mesenchymal stem cell pre-migration via the TLR2 pathway, and protect the regular intestinal cavity, its effect on the preservation of transplanted tumor tissue is minimal (387). Additional research has revealed that the radioprotective properties of the microflora are mediated by SCFAs, particularly propionate, and specific tryptophan metabolites generated by the microbiota (377). These results offer a possible therapeutic target for reducing radiotherapy-related side effects and alleviating radiation-induced harm (**Table 2**).

**Table 2:** A summary of microbiomes and their role in the treatment of GI cancers.

Type of treatment	Methods Used	Conclusion	References
Immunotherapy	N/A	- The success of cancer immunotherapy is influenced by microbiota. - The harnessing of microbiota has the potential to enhance the body’s immune response against tumors, thereby promoting antitumor immunity.	Goc et al. (378)
	-High-throughput sequencing technology -Regulation of gut microbiota	- The intestinal microbiota plays a crucial role in the progression and control of GI cancer. - The regulation of gut microbiota is suggested as a novel approach for treating GI issues.	Liu et al. (379)
	N/A	- The dysbiosis of the gut microbiome has an impact on both the prognosis and treatment of tumors. - The microbiota can enhance the anti-cancer immune response.	Wan et al. (380)
	- Meta-analysis was conducted on 16S rRNA gene sequencing data. - A multivariate selbal analysis is employed in order to identify bacterial genera.	- Gut microbiome features may predict immunotherapy response. - The application of machine learning algorithms has the potential to enhance the prognosis of cancer patients.	Liang et al. (381)
	-recruited 74 patients with advanced gastrointestinal cancer receiving anti-PD-1/PD-L1 therapy and collected stool samples before and during immunotherapy, along with clinical evaluations. -16S rRNA taxonomy survey	- The potential of the microbiome as a marker for immune-checkpoint blockade responses is indicated by the impact of gut microbiomes on anti-PD-1/PD-L1 outcomes, particularly in a subset of GI cancer patients.	Peng et al. (382)
Chemotherapy/immunotherapy/ radiotherapy	- The identification of particular gut microorganisms for use as biomarkers is being investigated through screening processes. -Fine-tuning the gut microbiota for cancer prevention	- Gut microbiota’s role in cancer development is crucial. - Improving cancer treatment outcomes can be achieved by adjusting gut microbiota through fine-tuning.	Zhou et al. (383)
Chemotherapy	-The association between *F. nucleatum* and chemotherapy response was investigated in 120 ESCC resected specimens and 30 pre-treatment biopsy specimens.	- *F. nucleatum* induces chemoresistance in ESCC cells through the regulation of autophagy. - Targeting *F. nucleatum* during chemotherapy could lead to different therapeutic results for patients with ESCC.	Liu et al. (384)
	- Genes that are differentially expressed in colorectal cancer cell lines due to infection by *F. nucleatum* were examined using a comprehensive analysis of the entire genome via microarray. - examined the clinical significance of *F. nucleatum* infection, BIRC3 protein expression, and resistance to 5-Fu treatment in patients with CRC.	- *F. nucleatum* and BIRC3 have the potential to be effective therapeutic targets in combating chemoresistance to 5-Fu treatment in advanced CRC.	Zhang et al. (385)
Radiotherapy	Three cohorts of patients (n = 134) were recruited The early cohort (n = 32) The late cohort (n = 87) The colonoscopy cohort compared the intestinal mucosa microenvironment in patients with radiation enteropathy (cases, n = 9) with healthy controls (controls, n = 6)	- The microbiota offers potential for the anticipation, avoidance, or management of radiation enteropathy.	Reis et al. (386)
	- Intestinal radioprotection was simulated through the use of cell lines and enteroids *in vitro*, and through the assessment of clinical outcomes and crypt survival *in vivo*. - The study utilized fractionated abdominal radiation and a single dose of radiation, in combination with syngeneic CT26 colon tumor grafts, to evaluate the efficacy of tumor radioprotection.	*-Lactobacillus rhamnosus* GG (LGG) functions as a controlled-release vehicle, delivering lipoteichoic acid with radioprotective properties. - lipoteichoic acid initiates a multi-step immune response involving macrophages and PGE2-secreting MSCs to safeguard epithelial stem cells within the stem cell niche.	Riehl et al. (387)

N/A, No Answer.

## Conclusion

Gastrointestinal (GI) cancer constitutes one of the new cancer cases worldwide and imposes a significant burden on public health, thus presenting a major threat to human population health. Disturbances in the gastrointestinal microbiota may have a significant impact on the development of gastrointestinal cancers. Some bacteria have been found to support the development of cancer, while others appear to protect against it. Studies have shown that changes in the composition and abundance of microbiomes can be associated with the development of various gastrointestinal cancers, such as colon, stomach, liver, and esophageal cancers. In this study, we examine the importance of gut microbiomes in gastrointestinal cancers and their impact on various gastrointestinal cancer treatments, including chemotherapy, immunotherapy, and radiotherapy. The information in this article paves the way for researchers in the field of cancer and microbiome.

## Author contributions

AY: Data curation, Writing – original draft. HA: Methodology, Project administration, Supervision, Writing – review & editing.
